# New approach methodologies in human regulatory toxicology – Not if, but how and when!

**DOI:** 10.1016/j.envint.2023.108082

**Published:** 2023-07-04

**Authors:** Sebastian Schmeisser, Andrea Miccoli, Martin von Bergen, Elisabet Berggren, Albert Braeuning, Wibke Busch, Christian Desaintes, Anne Gourmelon, Roland Grafström, Joshua Harrill, Thomas Hartung, Matthias Herzler, George E.N. Kass, Nicole Kleinstreuer, Marcel Leist, Mirjam Luijten, Philip Marx-Stoelting, Oliver Poetz, Bennard van Ravenzwaay, Rob Roggeband, Vera Rogiers, Adrian Roth, Pascal Sanders, Russell S. Thomas, Anne Marie Vinggaard, Mathieu Vinken, Bob van de Water, Andreas Luch, Tewes Tralau

**Affiliations:** a German Federal Institute for Risk Assessment (BfR), Berlin, Germany; b National Research Council, Ancona, Italy; c Helmholtz Centre for Environmental Research-UFZ, Leipzig, Germany; d German Centre for Integrative Biodiversity Research (iDiv) Halle-Jena-Leipzig, Leipzig, Germany; e University of Leipzig, Faculty of Life Sciences, Institute of Biochemistry, Leipzig, Germany; f European Commission, Joint Research Centre (JRC), Ispra, Italy; g European Commission (EC), Directorate General for Research and Innovation (RTD), Brussels, Belgium; h Organisation for Economic Cooperation and Development (OECD), Environment Directorate, Paris, France; i Karolinska Institute, Solna, Sweden; j Center for Computational Toxicology and Exposure (CCTE), United States Environmental Protection Agency (US EPA), Durham, USA; k Center for Alternatives to Animal Testing (CAAT), Johns Hopkins Bloomberg School of Public Health Baltimore MD USA, CAAT-Europe, University of Konstanz, Konstanz, Germany; l European Food Safety Authority (EFSA), Parma, Italy; m NTP Interagency Center for the Evaluation of Alternative Toxicological Methods (NICEATM), National Institute of Environmental Health Sciences (NIEHS), Durham, USA; n CAAT‑Europe and Department of Biology, University of Konstanz, Konstanz, Germany; o Centre for Health Protection, National Institute for Public Health and the Environment (RIVM), Bilthoven, the Netherlands; p NMI Natural and Medical Science Institute at the University of Tuebingen, Reutlingen, Germany; q SIGNATOPE GmbH, Reutlingen, Germany; r Wageningen University and Research, Wageningen, the Netherlands; s European Partnership for Alternative Approaches to Animal Testing (EPAA), Procter and Gamble Services Company NV/SA, Strombeek-Bever, Belgium; t Scientific Committee on Consumer Safety (SCCS), Vrije Universiteit Brussel (VUB), Brussels, Belgium; u F. Hoffmann-La Roche Ltd, Basel, Switzerland; v Fougeres Laboratory, French Agency for Food, Environmental and Occupational Health and Safety (ANSES), Fougères, France France; w National Food Institute, Technical University of Denmark, Lyngby, Denmark; x Vrije Universiteit Brussel (VUB), Brussels, Belgium; y Leiden University, Leiden, the Netherlands

**Keywords:** New approach methodologies, Chemical safety, Human health protection, Regulatory risk assessment, Next generation risk assessment, Regulatory toxicology

## Abstract

The predominantly animal-centric approach of chemical safety assessment has increasingly come under pressure. Society is questioning overall performance, sustainability, continued relevance for human health risk assessment and ethics of this system, demanding a change of paradigm. At the same time, the scientific toolbox used for risk assessment is continuously enriched by the development of “New Approach Methodologies” (NAMs). While this term does not define the age or the state of readiness of the innovation, it covers a wide range of methods, including quantitative structure–activity relationship (QSAR) predictions, high-throughput screening (HTS) bioassays, omics applications, cell cultures, organoids, microphysiological systems (MPS), machine learning models and artificial intelligence (AI). In addition to promising faster and more efficient toxicity testing, NAMs have the potential to fundamentally transform today’s regulatory work by allowing more human-relevant decision-making in terms of both hazard and exposure assessment. Yet, several obstacles hamper a broader application of NAMs in current regulatory risk assessment. Constraints in addressing repeated-dose toxicity, with particular reference to the chronic toxicity, and hesitance from relevant stakeholders, are major challenges for the implementation of NAMs in a broader context. Moreover, issues regarding predictivity, reproducibility and quantification need to be addressed and regulatory and legislative frameworks need to be adapted to NAMs. The conceptual perspective presented here has its focus on hazard assessment and is grounded on the main findings and conclusions from a symposium and workshop held in Berlin in November 2021. It intends to provide further insights into how NAMs can be gradually integrated into chemical risk assessment aimed at protection of human health, until eventually the current paradigm is replaced by an animal-free “Next Generation Risk Assessment” (NGRA).

## 1. Introduction

On November 15–17, 2021, the German Federal Institute for Risk Assessment (BfR) hosted the public symposium “Challenges in Public Health Protection in the 21st Century: New Methods, Omics and Novel Concepts in Toxicology”, where the latest developments regarding the use of NAMs in the regulatory context were presented. The symposium was followed by a workshop in which invited leading experts in the field systematically analyzed the technological readiness level as well as conceptual and regulatory hurdles hampering increased application of NAMs for the benefit of chemical safety and human health protection. In addition, experts presented their personal vision for how a primarily NAM-based system of chemical risk assessment should look in the (near) future. The two events were held as part of BfR’s ongoing effort – together with its international partner institutions - to promote scientific progress in the field of regulatory toxicology (Marx-Stoelting et al. 2015; Riebeling et al. 2018; Scholz et al. 2022; Tralau et al. 2015; Tralau et al. 2021).

To realistically assess the potential risk to human health posed by exposure to chemicals, the most relevant information related to possible adverse effects and exposure in humans should be used. Nevertheless, it is increasingly debated whether such fundamental aspects of chemical risk assessment have been adequately considered in today’s chemicals regulation. Relying on hazard data from experimental animals treated up to their maximum tolerated dose comes with inherent uncertainty about risks to be expected at realistic human exposure levels under foreseeable scenarios and raises the all-important question of human relevance both in qualitative and quantitative terms. On the other hand, the animal-centric approach has, so far, served us well and has offered an overall high level of health protection. Also, the concept of using directly measured outcomes in an intact and complex mammalian organism to predict effects in humans is reasonable in principle. Animal testing has been the cornerstone of chemical risk assessment for decades. Yet, why do many in the regulatory community see a need for change?

### 1.1. Shortcomings of traditional animal testing in chemical risk assessment

The current approach of chemical safety assessment is confronted with a number of challenges. In Europe, Regulation (EC) 1907/2006 on the Registration, Evaluation, Authorization and Restriction of Chemicals (REACH) entered into force in June 2007; 16 years later, it has led to the creation of the most comprehensive chemical safety-related database in the world. However, for a large share of the thousands of chemicals on the European market, data relevant for a meaningful and sound risk assessment are still lacking or incomplete. For higher-tonnage chemicals with more stringent information requirements, this can often be attributed to a combination of inadequate compliance on the side of the registrants, insufficient understanding of the concept and pre-requisites of read-across or a lack of assessment and enforcement capacities on the authorities’ side (ECHA, 2019; ECHA, 2020a; ECHA, 2021a). At the same time, a comprehensive analysis of the REACH registration by means of machine learning technology allowed the identification of reproducibility issues of the eight most common acute and topical OECD guideline tests (Luechtefeld et al. 2018). In addition, for chemicals marketed at a low annual tonnage by individual registrants (which may nevertheless account for a higher marketed tonnage overall), the regulatory information requirements in place only include a basic set of indicators for judging whether registered uses might pose a risk. Putting aside ethical concerns as well as questions about the scientific validity of the current paradigm, it would likely take a decade or more as well as a vast amount of testing resources/capacities to fully complete the REACH database according to the current information requirements of the REACH Annexes VII – XI. Conducting animal tests simply takes too long and is too expensive to be carried out for the sheer number of chemicals that are currently on the market and those anticipated to enter it in the years to come. These performance issues of the existing system alone seem justification enough to consider a fundamental change of paradigm. For new chemicals and product development, similarly, the resources and time needed for animal-based assessments often do not meet the time-to-market needs of the industry. Hence, there is a clear need to accelerate and increase the efficiency of toxicological data generation, data analysis of marketed chemicals and risk assessment; the first task, however, is neither achievable nor desirable using traditional animal testing.

In addition to these general considerations and ethical concerns, an increasing number of chemicals (Fischer et al. 2020), novel materials (e. g. nanomaterials) (Doak et al. 2022), or new areas of concern (developmental neurotoxicity (DNT), immunotoxicity, endocrine disruption (ED)) (Lupu et al. 2020; Sabuz Vidal et al. 2021) challenge the current system. Moreover, the performance of traditional rodent tests, widely perceived as the “gold standard” for e.g. repeated-dose toxicity and carcinogenicity, has been called into question (Goodman 2018; Luijten et al. 2020; Smirnova et al. 2018). Shortcomings regarding interspecies concordance between different mammalian or even rodent species, as well as with respect to extrapolation from experimental animals to humans, the ambiguity of results or poor reproducibility performance cast doubts on the relevance of such test methods for human health risk assessment (Browne et al. 2018; Karmaus et al. 2022; Kleinstreuer et al. 2016; Luechtefeld et al. 2016; Pham et al. 2020; Wang and Gray 2015).

Mixture risk assessment (MRA), i.e. the assessment of potential adverse effects due to combined exposure to multiple substances, is another regulatory area where the current animal-centric paradigm fails to provide adequate solutions. Regulatory authorities such as EFSA and US EPA carry out MRAs regularly during risk evaluation of selected chemicals, interrelated groups of chemicals or mixtures based on animal data (e.g. EFSA 2020; US EPA 2017). However, a robust MRA ideally builds on detailed knowledge of the toxicological mechanism (e.g. molecular target(s)) of all co-exposure-forming substances (EFSA 2021). Such mechanistic insights, however, are usually not available from traditional *in vivo* studies adding uncertainty to the assessment. On the other hand, testing large numbers of potentially harmful mixtures in experimental animals is certainly not feasible (de Jong et al. 2022; Tralau et al. 2021).

With that in mind, it becomes clear that today’s animal-centric approaches of hazard identification and risk assessment will require significant innovation over the coming years to ensure sustainability as well as rapid and effective responses to the challenges posed by chemicals of the future. This, together with stronger policy, financial and regulatory support, is also envisioned by the Chemicals Strategy for Sustainability (CSS) (European Commission 2020), even though open scientific questions remain in this respect (Barile et al. 2021; Batke et al. 2022; Herzler et al. 2021, Herzler et al. 2022; Scholz et al. 2022). At the same time, one should be aware that any change will have to fit into the current regulatory framework at least in general terms. Given the complexity and interoperability of the underlying legal requirements, proposals for changes with regard to testing or assessment need to be designed in a way that there is scientific as well as regulatory and legal compatibility. Of note, this also holds true for the predefined requirements of risk management.

Therefore the present paper predominantly focuses on the hazard part of chemical risk assessment, while acknowledging that the exposure part clearly also needs continuous methodological revisions. More and more data on use and exposure as well as new methods for their integration into risk assessment become available, with the ultimate goal of providing tailored data to optimally inform differentiated risk management decisions.

### 1.2. NAMs as replacement, reduction and refinement of traditional animal testing

**Fig. 1.**
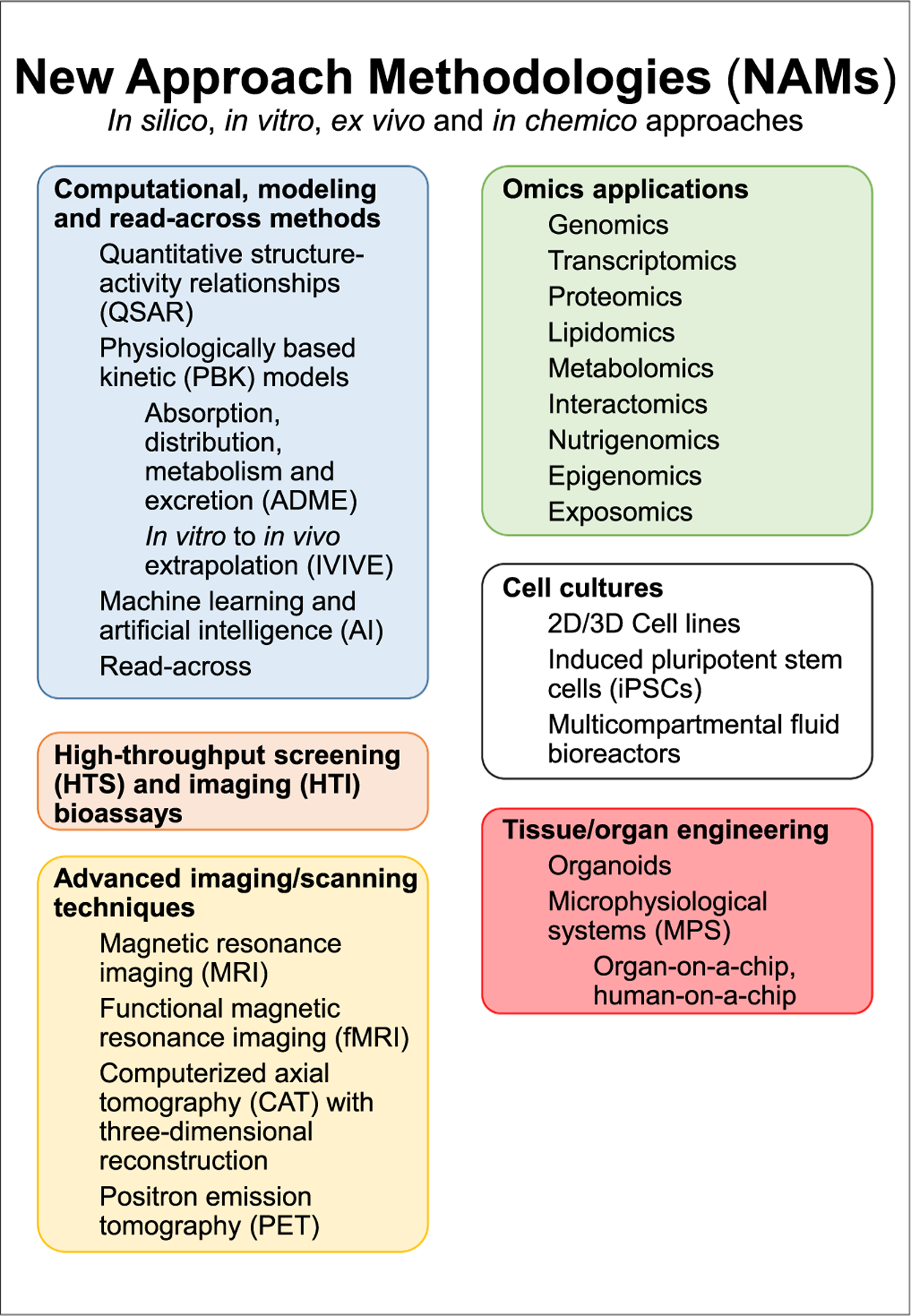
List of methods, techniques, tools, applications and systems commonly encompassed under the umbrella of NAMs.

To overcome some of the above-mentioned shortcomings, an increasing number of NAMs have been developed over the past years with the ultimate goal of providing a system that is scientifically better, more efficient and more ethical. NAMs can generally be described as alternative or complementary methods to or an enhancement of traditional animal testing to predict hazardous properties of chemicals. The development of NAMs and their use for risk assessment follows the 3R principles of replacing, reducing, and refining standard animal experiments. Such methods/test systems include high-throughput screening (HTS) bioassays, the field of omics applications, cell cultures, organoids and other microphysiological systems (MPS) (Marx et al. 2020; Marx et al. 2016), machine learning models and artificial intelligence (AI), quantitative structure–activity relationship (QSAR) predictions, and read-across (Escher et al. 2019; Rovida et al. 2020) (Fig. 1).

Generally, NAMs can be broken down into *in silico*, *in chemico*, and *in vitro* methods where *in silico* approaches are computational tools aiming at modelling endpoints such as toxicokinetics (Thompson et al. 2021) or metabolism (Leonard 2019), or at predicting effects based on chemical structural features and association with legacy data; *in chemico* is a general term referring to the use of abiotic chemical reactivity methods (Gerberick et al. 2008); *in vitro* data generated using human cells or organoids may be used to directly inform cellular targets and chemical-induced molecular mechanisms potentially leading to adversity, which can be conceptually organized using Adverse Outcome Pathways (AOPs) (Ankley et al., 2010; Chauhan et al., 2021; Leist et al., 2017; OECD, 2021a). Omics technologies enable insights into a broad spectrum of complex biological responses triggered by chemical perturbation (Hartung and McBride 2011). Thus, unlike traditional toxicological tests generating knowledge on apical adverse outcomes in experimental animals, NAMs deliver evidence as to why an adverse outcome is likely to occur and enable systems toxicology (Hartung et al. 2017; Hartung et al. 2012). For well-understood AOPs, this helps to better inform the decision of whether a certain apical effect observed in an animal test should be considered relevant for humans. The concept is essential in identifying groups of substances causing the activation of identical or converging AOPs (either individually or networks), as a prerequisite for regulatory grouping and for mixture risk assessments (Beronius et al. 2020; Rotter et al. 2018).

NAMs not only serve as alternative non-animal approaches but can also be combined with *in vivo* test methods, *in vitro* studies and clinical observations to ultimately build/expand AOPs (Ball et al. 2022; Caloni et al. 2022; Leist et al. 2014). Gathering omics data within a short-term rodent bioassay, for instance, provides insights into pathways of disturbance and facilitates decision-making based on experiments with fewer animals or shorter duration (Gwinn et al. 2020; Thomas et al. 2011; Thomas et al. 2013b). When incorporated into animal test designs, NAMs can provide mechanistic insights, and when human-based, they can ascertain the human relevance of a response; in both cases, they ultimately enhance the predictive capacity.

Chemical risk assessments were successfully conducted without leveraging animal data for several endpoints, e.g., in Clerbaux et al. (2019); Ramanarayanan et al. (2022). Several NAMs for a variety of endpoints such as local toxicity (skin sensitization, skin/eye irritation/corrosion, phototoxicity), dermal absorption, mutagenicity/genotoxicity, as well as quality control measures such as pyrogenicity, are fit for regulatory purposes, their value is increasingly recognized by, e.g., the OECD and accordingly included as information source into IATA frameworks. Skin sensitization, for instance, has been thoroughly addressed by OECD and, in addition to animal-based test guidelines (TGs), three mechanistically-based *in chemico* and *in vitro* test methods – TGs 442C (OECD 2022c), 442D (OECD 2022d) and 442E (OECD 2022e) - were established to inform on the hazard potential of chemicals with respect to the first three key events of the corresponding AOP. These were later incorporated in the OECD TG 497 on Defined Approaches (DAs) for Skin Sensitization (OECD, 2021d), demonstrating that the limitations of any single *in vitro* method can be overcome by integrating several NAMs.

Preliminary chemical screening and prioritization (Luijten et al. 2022), the generation of data to support read-across (Ball et al. 2020), or data gap filling are areas where NAM-based data are commonly utilized by industry and regulatory authorities, at least for internal usage (Escher et al. 2019; Rovida et al. 2020; van der Stel et al. 2021). Screening data generated with an extensive battery of *in vitro* assays for an impressive number of chemicals under ToxCast (https://www.epa.gov/chemical-research/exploring-toxcast-data-downloadable-data) and Tox21 (https://tox21.gov/resources/) are widely used and appreciated by the regulatory community, e.g., Endocrine Disruptor Screening Program (EDSP) data for mechanistic considerations within the European pesticides regulation. The project “Accelerating the Pace of Chemical Risk Assessment” (APCRA) started in 2016: this governmental initiative aims to promote collaboration on scientific and regulatory needs to advance the regulatory acceptance of NAMs (Kavlock et al. 2018). In its effort to reduce the use of vertebrate animals for toxicity testing, the US EPA has developed a work plan to prioritize resources towards NAM-related activities (US EPA 2021). Over the past years, several European projects have also explored the capacity of NAMs in the regulatory context, including ReProTect (2004–2009) (Hareng et al. 2005), the SEURAT-1 cluster (2011–2016) (Daston et al. 2015; Gocht et al. 2015), EuroMix (2015–2019) (Rotter et al. 2018), and the recently-concluded EU-ToxRisk (2016–2022) (Moné et al. 2020). In 2021, PANORAMIX, a project on the NAM-based risk assessment of complex real-life mixtures, kicked off (Escher et al. 2022). Additional projects initiated within the EURION and ASPIS clusters are summarized in Supplementary Table 1. Recently, the European Partnership for Alternative Approaches to Animal Testing (EPAA) started a project that brings together scientists from industry and the EU Commission to collaboratively work on NAM-based case studies (Westmoreland et al. 2022). When critically evaluating the challenges these projects have faced, some recurring issues can be identified. This includes that a larger involvement of regulators in such projects is required to advance the uptake of NAMs into regulatory processes. Additionally, the high level of standardization, validation and training that is needed when implementing NAMs into risk assessment has been highlighted. The storage and availability of data from EU projects to the regulatory community have been identified as critical aspects as well. Also, even though funding was sufficient to achieve the scope of the listed projects to fully implement NAM-based ab initio testing, more funding is needed. Consequently, the European Partnership for the Assessment of Risks from Chemicals (PARC) initiative, which kicked off in May 2022, addresses these critical points as not only major funding has been provided to foster innovation and to accelerate the regulatory acceptance of NAMs but also the inclusion of regulators in the management of the project, standardization, communication with policy, application of FAIR principles to data management and training have been addressed. For an overview on PARC see Marx-Stoelting et al. (2023) or https://www.eu-parc.eu.

## 2. Challenges for the implementation of NAMs in the regulatory system

**Fig. 2.**
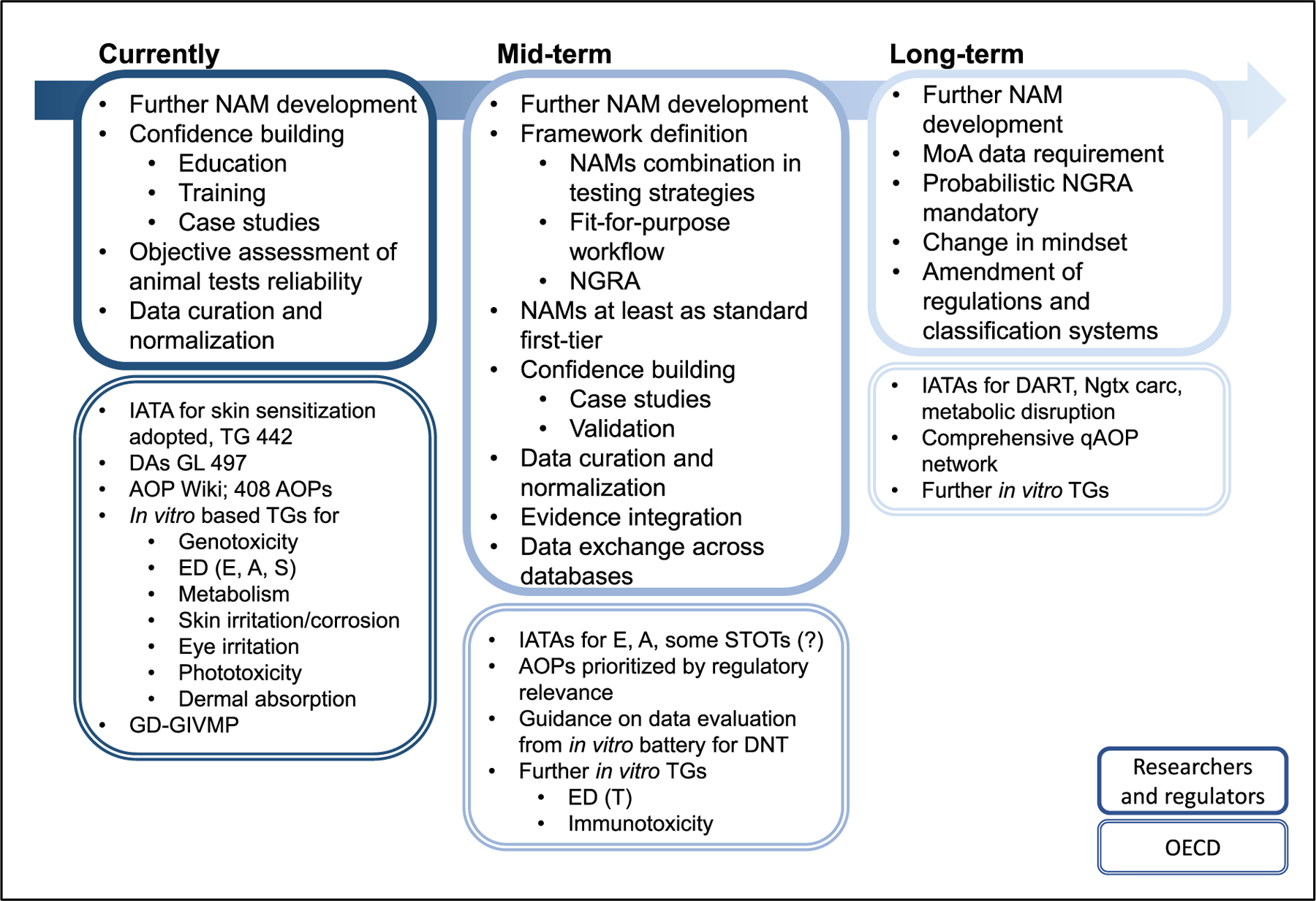
Tentative timeline of the actions envisioned for overcoming limitations preventing NAM implementation into regulatory toxicology. Timeframe and stakeholder responsibilities are indicated by box outline color shading and type, respectively. NAMs established as OECD test guidelines are included in the bottom left box. TG: test guideline; DAs GL: guideline for defined approaches; ED: endocrine disruptors; GD-GIVMP: guidance document on good *in vitro* method practices; STOTs: specific target organ toxicities; DNT: developmental neurotoxicity; DART: development and reproductive toxicology; Ngtx carc: non-genotoxic carcinogens.

Despite the tremendous and continuous effort put into the development of NAMs in the last decade, the pace of their implementation in regulatory toxicology remains slow. Data produced by using NAMs on their own are currently not perceived by the regulatory community as sufficient to conclude on a broad spectrum of chemical safety-related endpoints for plant protection products, industrial chemicals, cosmetics or pharmaceuticals. In the following sections, some of the obstacles in the way of regulatory NAM implementation are addressed in more detail along with an account of the progress that has been made so far in trying to overcome them (Fig. 2).

### 2.1. Current paradigm of chemical risk assessment

One of the major reasons for the slow uptake of NAMs into the regulatory processes lies in the way in which the legislation implements the current animal-centric paradigm. The standard information requirements (SIRs) for the more complex apical endpoints under REACH, for instance, stipulate specific animal test designs for hazard identification and characterization. This approach provides clarity and legal certainty for the stakeholders involved. In addition, adaptation of the SIRs by using alternative methodology is possible in principle. Annex XI of the REACH legislation specifies that alternative methods need to be “adequate for the purpose of classification and labelling and/or risk assessment” (i.e. allow for quantitative hazard characterization). However, this is challenging especially for the more complex endpoints such as repeated-dose and reproductive toxicity or carcinogenicity. Employing (a set of) NAMs to comply with the SIRs for a sub-chronic 90-day toxicity study, for instance, would require the generation of comparable information generally obtained from the standard animal study (OECD TG 408). The coverage of all organs/systems and key parameters of a TG 408 study along with sub-chronic 90-day exposure duration, and the possibility to derive a reliable quantitative “Point of Departure” (PoD) are essential requirements. At best, one could hope to address them by means of a battery approach, i.e. using several NAMs in concert, each monitoring a key event in the underlying AOPs. However, a registrant submitting such a battery approach would also have to demonstrate how its results are adequate for classification and labelling. For complex endpoints, this, however, is virtually impossible, since the current criteria for classification and labelling under the Globally Harmonized System of the United Nations (UN GHS) and its European implementing Regulation (EC) 1272/2008 (CLP Regulation) relate to data either obtained in humans *in vivo* or from animal tests according to the established OECD/EU Test Method guidelines or other internationally accepted test methods. Although a time-consuming process, these regulations would need to be revised to also include criteria applying NAM-based battery approaches. In addition, the overlap of the different systemic toxicity endpoints must be thoroughly understood, and which NAM is fit for the purpose to inform about adverse outcomes identified by current endpoints must be clarified.

This focus on classical animal experimental designs greatly hampers the flexibility for developing and implementing NAMs. For example, a sequential NAM battery design, first screening broadly and qualitatively for organ/systems toxicity, following up only on positive findings and deriving a quantitative PoD for effects considered plausible and relevant would currently not be accepted by ECHA under REACH. Overall, this results in the paradox that although animal testing is nominally described as a last resort under REACH, the traditional animal test designs are the first and last reference points for registrants. In addition, the industry, even if willing to enter new ground, is not incentivized to do so due to the high economic risk of rejection (in the case of risk characterization) or possible legal liabilities (in the case of self-classification).

The focus on the apical systemic toxicity endpoints in the classical animal tests also has another implication detrimental to the implementation of NAMs in regulatory frameworks. Most NAMs provide a readout at the molecular, genomic, transcriptomic, proteomic or cellular level. As such, they can be indicators of downstream apical effects at the organism level, but they cannot show such effects directly unless properly validated. To establish trust in their predictive reliability, additional proof of qualitative (e.g. via AOP networks), quantitative (e.g. by quantitative AOPs (qAOPs) and quantitative *in vitro* to *in vivo* extrapolation, QIVIVE) and temporal coherence with apical outcomes observed *in vivo* is required. Because essential elements of a whole organism (e.g. blood and lymphatic circulation, intra-organ cell diversification and communication) are missing in most single NAM, a complex network of different NAMs would be needed to reproduce an *in vivo* 90-day repeated-dose toxicity test in all relevant aspects. Not only might this complexity be an additional disadvantage compared to the classical animal tests (which can currently be commissioned to a contract research facility at a calculable price), but adapting the regulation to allow for the use of such complex and currently difficult-to-validate NAM batteries/networks would also be a highly resource- and time-intensive effort in the first place (Hartung et al. 2013c; Rovida et al. 2015). Worthy of note, a risk assessment performed by means of NAMs and PBK models may not necessarily be cheaper than one carried out via traditional methods, and this is also not a requirement per se, but it has indeed the potential to be more informative and more human-relevant.

To avoid extensive animal testing, REACH Annex XI also opens up the possibility for registrants to adapt SIRs by utilizing grouping and read-across (European Commission 2006). Albeit the most frequently applied alternative method to fill existing data gaps, these non-testing approaches are often rejected by ECHA for a lack of thoroughness and rigor (Ball et al. 2016; ECHA 2020b). Additional NAM-based data may not only increase the regulatory acceptance of grouping and read-across approaches, but would enable authorities to screen and prioritize more efficiently. In fact, the importance of NAM data inclusion in read-across applications particularly to address chronic endpoints has been recently highlighted, not only to strengthen read-across arguments but also to gain experience with the NAMs applied (Daston et al. 2022; Escher et al. 2019; Mahony et al. 2020). Likewise, a NAM-supported read-across approach (Zhu et al. 2016) is proposed as an initial starting point in a predictive toxicity framework published by the US National Academy of Sciences. The ultimate goal, thereby, is to advance the methodology to a point where measured data from analogues are not necessarily required anymore to predict the toxicity of an unknown chemical (NASEM 2017).

While the European regulatory system gains its protective strength from its rigorous, but inherently less flexible approach based on SIRs, the regulatory system in the US – with less rigid information requirements leading to less *in vivo* data - is inherently more flexible when it comes to the use of NAMs. As an example, various DAs (i.e. defined combinations of information sources interpreted using a fixed data interpretation procedure (DIP) to satisfy a specific regulatory need) are accepted by the US EPA and FDA agencies for a wide range of compounds such as pesticides, industrial chemicals, drugs, and biologicals (US EPA 2018; US FDA 2020). To the contrary, their use in Europe is currently still limited to the OECD-approved DAs for skin sensitization (Guideline No. 497) and serious eye damage and eye irritation (Guideline No. 467).

### 2.2. Is a general skepticism towards the use of NAMs in regulatory risk assessment justified?

Although substantial progress has been made in recent years, a general skepticism as to whether NAMs are sufficient to assure chemical safety continues to be a major obstacle for the implementation of NAMs into regulatory practice. Self-evidently, each single NAM has its limitations. Due to the lack of organismal complexity, missing toxicokinetic context, and the limitations in terms of treatment duration and toxicological space covered, doubts have been raised as to whether especially systemic toxicity and chronic toxicity endpoints can be successfully addressed by NAMs. Focusing on mechanistic information on early events such as Molecular Initiating Events (MIEs) or Key Events (KEs) in a complex network of pathways that may or may not result in an apical adverse outcome comes with a level of uncertainty that many regulators feel uncomfortable with. Moreover, testing less readily or insoluble substances using NAMs requires extended technical efforts, adding to the inherent limitations of the approach: it must be noted, though, that certain physico-chemical properties also impair animal testing. Hence, a comprehensive comparison of such substances *in vivo* versus *in vitro/in silico/in chemico* is strongly suggested to reveal whether the limitations only pertain to NAMs. Altogether, this results in a general mistrust towards NAMs, which has hampered progress towards their implementation so far.

Numerous NAMs have been developed so far, but risk assessors, and even more so risk managers, are often not sure what these methods can deliver. What information can be gained from an individual test and in which context is this information useful (e.g. prioritization, screening, or risk assessment)? Does the applicability domain of a certain assay cover the chemical space needed in a screening approach? Is the degree of certainty in the prediction sufficient to apply the method in definitive quantitative risk assessment? The fitness for purpose of NAMs in relation to a specific context of use is often unclear. The US Strategic Roadmap for Establishing New Approaches to Evaluate the Safety of Chemicals and Medical Products (ICCVAM 2018) emphasizes the necessity of defining the context of use of a NAM and using flexible and efficient validation approaches that are fit for the defined purpose.

Yet, for risk assessment regarding a number of endpoints (e.g. local toxicity), well-established and even validated NAMs (Leist et al. 2012) can already be employed and fully replace their traditional counterparts. Skin sensitization, for instance, can be assessed solely by a combination of non-animal testing strategies (OECD 2022c; d; e) with comparable performance to that of the animal or human data traditionally used for classification (Kleinstreuer et al., 2018; OECD, 2021d).

A prerequisite for the success of NAMs, from a predictive perspective, is a thorough understanding of the molecular mechanisms defining a certain adverse outcome both at cellular and organ level. Skin sensitization has been intensively investigated by the OECD Expert Group developing the OECD TG 497, and sufficient knowledge was generated to enable the development of such fit-for-purpose applications; this is not yet the case for most traditional apical endpoints, though, and the mechanistic understanding of many adverse effects or even chemically induced human diseases is largely incomplete. The assessment of the impact of chemicals on human health in large cohort studies is only progressing at a slow pace due to inherent limitations with regards to sampling, chemical assessment of the complexity of the exposure and statistical methods supporting the link between exposure itself and a specific effect. While the interest in exposomics has laid the path for large EU projects that tackle at least some of the challenges (e.g. the human exposome network: https://www.humanexposome.eu/) it will require additional work before one would routinely use these data in a regulatory context.

If the biology behind an effect is not clear, how can one develop a test method to address the effect? While for a whole organism-based animal test, a black box scheme might seem acceptable (but comes with considerable uncertainty, too), this is not the case for NAMs relating to a single KE in an AOP or providing a single readout of cell physiology. Nevertheless, the strength of an AOP lies in the dynamicity with which toxicity mechanisms are represented. While they can serve as an anchoring framework for risk assessment purposes, AOPs are also constantly updated as soon as new knowledge is generated from experimentation, mechanistic models, big data and predictive analyses. Such an approach is therefore iterative and mutually informative, and the ultimate goal is to provide insights into human toxicity mechanisms and their susceptibility to chemical perturbation. Currently, 435 AOPs, 759 prototypical stressors, 1671 KEs and 2434 KEs relationships are covered, at least partly, in the AOP Wiki (metrics as per March 17, 2023; https://aopwiki.org/). Twenty-nine AOPs are currently endorsed by the Working Group of the National Coordinators of the TG Programme (WNT) or the Working Party on Hazard Assessment (WPHA), while 15 are under review by the Extended Advisory Group on Molecular Screening and Toxicogenomics (EAGMST). A guidance document (GD) on quality standards required for the scientific review of an AOP on the AOP-Wiki portal was recently published (OECD, 2021a), building the foundation for an increased confidence in AOP quality on which their widespread acceptance as an information source to guide interpretation, generation and application of data from NAMs depends. It should also be noted that it is not always necessary to address all KEs of an AOP by testing, if measuring certain KEs by an accepted and validated (set of) NAM(s) has proven to be sufficiently predictive of the apical effect in humans, including quantitative aspects. The combined employment of multiple NAMs, though, needs to effectively distinguish between perturbational effects of an adaptive, temporary nature and an actual adverse effect (Dent et al. 2018). The potential of this approach is greatly exemplified by the development of an AOP-based Integrated Approach to Testing and Assessment (IATA) for DNT (Sachana et al. 2021). To further develop and improve the AOP framework, omics applications were found useful for e.g. refining existing or generating new AOPs (Brockmeier et al., 2017; van der Stel et al., 2021).

A chemical risk assessment may be conducted for a wide range of applications. For the purpose of risk characterization, a health-based guidance value (HBGV), i.e. a level of exposure that is considered safe for humans, needs to be derived by modelling dose-responses typically determined *in vivo* and applying assessment/uncertainty factors to extrapolate from the experiment or epidemiological study to the real-life exposure situation (WHO 2009). Many regulators currently consider NAMs as unable to provide quantitative data for HBGV derivation. Relying entirely on *in vitro* bioassays would require QIVIVE to determine a HBGV, but the applicability of existing QIVIVE approaches needs to be shown and extrapolation will, in any case, add another level of uncertainty (Wambaugh et al. 2018). On the other hand, a QIVIVE approach was recently published (Scholze et al. 2020) for which the added uncertainty may not be as big as that underlying the rodent-to-human extrapolation, but additional high-quality case studies are needed. The tiered NAM-based hazard evaluation strategy of the CompTox initiative at US EPA is oriented towards the estimation of PoDs for chemical perturbation of biology regardless of whether the biological target or pathway are lacking or defined. This provides an approach to utilize NAMs in a protective way, rather than requiring them to be predictive of a specific toxicity endpoint. The respective PoDs are estimated based on biological pathway or cellular phenotype perturbation, or based on AOPs or organotypic assays/microphysiological systems, respectively (Thomas et al. 2019). Dedicated software (e.g. BMD Express 2) or statistical packages (e.g. tcplfit2) have been developed that enable the calculation of quantitative PoDs by benchmark dose (BMD) modelling of omics data (Phillips et al. 2019; Sheffield et al. 2022; Yang et al. 2007).

Using qualitative NAM data within a margin of safety approach has been demonstrated for the risk assessment of cosmetic products by using PoDs based on *in vitro* assays together with exposure estimates (Baltazar et al. 2020). These examples show that creative solutions can be found to tackle specific challenges of NAM-based chemical risk assessment if regulatory frameworks allow for sufficient flexibility. Apart from risk assessment, the applicability of NAMs for screening and prioritization purposes has already been demonstrated (Rotroff et al. 2010). The European Chemicals Agency (ECHA), for instance, utilizes NAMs (e.g. QSAR models) in its Integrated Regulatory Strategy (IRS) to screen groups of chemicals for properties of concern in an attempt to prioritize each registered substance according to a possible need for regulatory action (e.g. data generation, regulatory risk management ongoing or under consideration, currently no further actions proposed) (ECHA 2021b). Worthy of note, according to a retrospective case study recently conducted as part of the APCRA project and co-authored by the ECHA Computational Assessment Unit, the vast majority of the 489 analyzed substances (89 %) had lower PoDs when based on high-throughput *in vitro* bioactivity as compared to traditional hazard information. While this may in part be due to the fact that NAMs often address early AOP events not necessarily leading to the adverse apical outcomes in the end, it was nevertheless demonstrated that, when combined with exposure predictions, NAM-based PoDs can be used for a conservative prioritization within a screening approach (Paul Friedman et al. 2020). Therefore, where the context of use is set to prioritizations, existing NAMs already have a significant potential. Moreover, to explore whether predictivity can be improved, an ongoing APCRA prospective case study is investigating whether a NAM battery can be employed to derive protective PoDs for systemic endpoints (i.e. 90-day repeated dose toxicity) in line with those obtained from traditional studies (Kavlock et al. 2018).

It is also commonly believed that demonstrating the absence of adverse effects is much easier with animal models than NAMs. Yet, a combination of various NAMs in a battery approach may be sufficient to determine biological activity or the absence of it. Analyzing, for instance, transcriptional responses, i.e., the entire range of transcriptional biological activity, to a chemical in an *in vitro* dose–response experiment may, in a weight of evidence approach, support that no chemical perturbation occurs at a molecular level in response to low doses. If, with a NAM battery, sufficient toxicological space is covered and experience with applying these techniques accumulates, the regulatory community will gain the confidence required to conclude that a given substance, in the absence of significant biological activity, could be considered less relevant for human safety. However, the generally higher sensitivity of NAMs would result in a system being protective, or even overprotective for humans. Therefore, following the initial over-classification of chemicals using NAM-based data, the regulatory community may be required to take exposure and potency into greater consideration and even re-define protection goals to effectively safeguard human health.

The general skepticism towards NAMs is, however, not just the result of the above-mentioned factors, but also often stems from a lack of familiarity with the technologies NAMs are based upon. Risk assessors have historically been trained to interpret results obtained from experimental animal studies. NAM approaches are often multi-/interdisciplinary in nature and require expertise in many different fields spanning from cell biology to bioinformatics. The scientific complexity behind NAMs is growing at an ever-increasing rate: this represents a two-fold challenge for risk assessors and managers in terms of the understanding and resources needed for adopting them, especially considering a global and diverse context. In this respect, we are confident that trust in and awareness of NAMs would increase by further exploiting and disseminating the knowledge generated within several ambitious EU-funded research projects (e.g., PARC, EU-ToxRisk, PANORAMIX - under the H2020 Green Deal call - and all projects within the EURION and ASPIS clusters- summarized in Supplementary Table 1) and constant efforts of regulatory agencies worldwide (Supplementary Tab. 1). This would require extensive training but also constant use of case studies via international bodies such as OECD to facilitate communication between regulatory and university scientists. This could also be a way forward towards greater standardization and harmonization of such methodology, which have proven to be key for worldwide acceptance in the regulatory arena.

It must also not be forgotten that animal data are associated with inherent variability and uncertainty as well. Indeed, direct comparison of variability *in vivo* and *in vitro* often will see animal systems perform less well than their alternative counterpart, the obvious reason for this being their inherent complexity. For instance, Luechtefeld and colleagues have claimed that the likelihood that a repeat test for eye irritation on the same chemical will return the same GHS sub-category is low when conducted via the classic rabbit eye irritation test, and mild or moderate irritants are actually more likely to be not classified if tested again than they are to actually reproduce their prior category (Luechtefeld et al. 2016). Depending on how data produced within a single traditional study are statistically modelled, the root-mean-square error (RMSE) can result in PoDs being up to 10-fold variable, meaning that a lowest observed adverse effect level (LOAEL) of 1 mg/kg body weight/day may actually range from 0.7 to 14 mg/kg body weight/day (Pham et al. 2020). Similar confidence intervals were observed when analyzing replicate acute oral systemic toxicity studies, where various chemical factors were investigated and found not to account for the inherent variability observed in the animal data (Karmaus et al. 2022). Liver toxicity is a major reason for drug withdrawal from the US market, indicating poor extrapolation from pre-clinical animal models to human clinical hepatotoxicity (Li et al. 2020). Of note, it is also unknown how many drugs never reached the market because of critical toxicity predicted by animal models that might not have been (quantitatively or even qualitatively) relevant for humans. Moreover, at least some health endpoints (e.g. some variants of endocrine disruption and some neurological diseases) are not well served by the current system either. Collaborative work among academia, industry and regulatory agencies is deemed necessary to incorporate a more transparent perspective on the uncertainty and variability in the animal reference data that is currently being used for hazard and risk assessment decisions. Wang and Gray (2015) evaluated 37 chemicals that had been tested in rodent bioassays in rats and mice of both sexes within the US National Toxicology Program for non-carcinogenic endpoints: little concordance between species and sexes was found. *“Overall, there is considerable uncertainty in predicting the site of toxic lesions in different species exposed to the same chemical and from short-term to long-term tests of the same chemical.”*.

Because of these inherent animal data limitations, the validation of NAMs should (at a minimum) be held to the same standard of variability as traditional methods and also be performed taking into account their relevance to human physiology and biological mechanisms as opposed to the performance of traditional animal test results (i.e. prediction of effects on human KEs) by default, even though this comparison has been successfully applied when considering weaknesses of animal models and species specificity (Lichtenstein et al. 2020). As human test data are typically not available, a concept of mechanistic validation has been proposed (Hartung et al., 2013b), which aims to demonstrate the coverage of relevant pathomechanisms.

## 3. Possible ways forward

Over the past decade, cell culture-based toxicological testing in combination with high-content visualization techniques, complex organoid systems, omics, advanced modelling and prediction systems have updated the toxicological toolbox profoundly. These scientific advances gave rise to an increased understanding of systems biology and toxicological mechanisms, leading to the development of new conceptual approaches to chemical hazard assessment. The use of NAM approaches also provided insights into the nature of possible interactions of chemicals with regard to their biological targets. This knowledge has significant implications as to how NAM-based testing may be constructed, what percentage of chemicals may benefit from the application of AOPs (e.g., if a chemical confers its bioactivity through rather nonselective interactions with cellular structures, then identifying the plethora of MIEs/KEs and associated AOPs is challenging and deriving more generic bioactivity based PODs may be more appropriate), and which KEs/MIEs are more frequent targets of environmental chemicals (Thomas et al. 2013a).

At the same time, thanks to the REACH Regulation, but also to the Tox21 federal research initiative in the US, an increasing number of toxicological data are available to the public domain through interfaces such as the Integrated Chemical Environment (https://ice.ntp.niehs.nih.gov/) and the EPA CompTox Chemicals Dashboard (https://comptox.epa.gov/dashboard/) (Thomas et al. 2018).

**Fig. 3.**
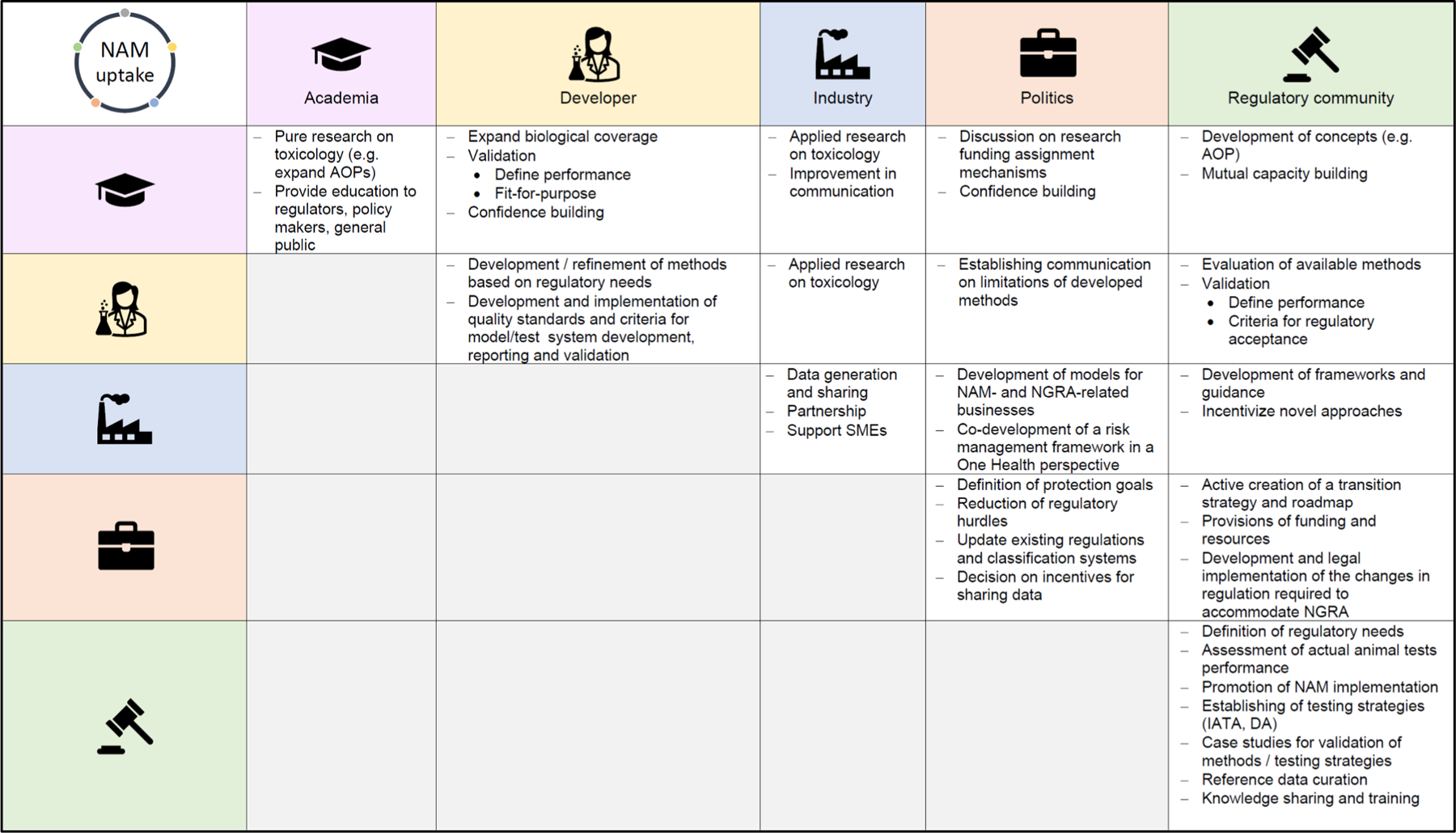
Key responsibilities of stakeholders involved in the regulatory transition from the traditional animal-based approach to a NAM-grounded system. Intersections of the triangular matrix indicate individual (e.g. academia-academia) and collaborative (e.g. academia-developer) stakeholder engagement. All actions are to be intended direction-free. Stakeholders were sorted alphabetically.

As science is not static, also regulatory systems grounded in science must take such new developments into account by frequently updating the regulatory toolbox to safeguard public health based on the latest scientific insights. To this end, a concerted effort from all stakeholders involved is advised. This is challenging as procedures for updating vary according to the stakeholders involved and due to the conceptual architecture of the underlying regulatory framework. A bottom-up approach to testing and assessment, for example, will inherently profit more from prioritization than a test-all top-down approach. Although there cannot be a one-fits-all solution, we propose the following overarching concept for the roles of the stakeholders involved (Fig. 3).

### 3.1. Testing and assessment strategies for single substances and mixtures

Notably, while some frameworks – particularly those in systems prone to operating bottom-up – already show remarkable flexibility with regard to the uptake of new methods, many others are by design less flexible. If sufficient understanding of an AOP network leading to a certain adverse outcome is available and depending on the context of use, information requirements can be defined, e.g. by assigning fit-for-purpose NAMs to the various components of the network such as MIEs and KEs. Likewise a sufficiently validated set of effect biomarkers might be available, for instance from the use of metabolomics-derived biomarkers linked to a certain adverse outcome (Taylor et al. 2018). In both cases, combining multiple NAMs in an IATA or DAs may subsequently provide sufficient information to confidently assess the likelihood of an adverse effect in humans. Basing such IATAs on AOP frameworks is particularly promising (Tollefsen et al. 2014). The importance of developing such integrated approaches has been greatly exemplified by the development of the OECD guideline 497 for DAs for skin sensitization (OECD, 2021d). Thereby, combinations of the most appropriate NAM-based information from multiple sources (*in chemico*, *in vitro* and *in silico*), mapped to the AOP, with standardized data interpretation procedures were established in strategies that have since successfully proven its potential to fulfil regulatory needs and to replace the traditional animal test. Having such a strategy at hand, one can examine its performance against reference data, characterize the applicability domain and evaluate the degree of uncertainty. Within a transition period, one could begin by employing simple, available and validated alternative tools in a tiered strategy with broad biological coverage type assays (e.g. transcriptomics and phenotypic profiles) and data mining as tier one for substance categorization, allowing developers to optimize the scientifically more challenging methods for systemic/long-term toxicity and regulatory confidence to grow. A tiered testing strategy combining *in silico* and *in vitro* approaches for hazard characterization has been proposed by the US EPA (Thomas et al. 2019). A recent review paper aimed at encouraging a learning-by-doing mentality and developing a robust, yet adaptive NAM-based approach (Nymark et al. 2020): 37 out of the 50 NAMs included in a tool package to support risk governance of nanomaterials were, for instance, considered ready for initial exploration.

Other initiatives are currently exploring strategies to further address human adverse outcomes with NAMs. For instance, the assessment of DNT is hindered for many substances by the unavailability of relevant data as a result of ineffective *in vivo* test methods (Smirnova et al. 2014). At the same time, there is a particular concern that chemicals may play a role in the etiology of neurodevelopmental disorders. To address the lack of data on a large number of substances, the regulatory community has been exploring NAM-based testing strategies. A collaborative effort coordinated by OECD is currently working towards the regulatory uptake of an *in vitro* battery for DNT (IVB DNT) as part of an AOP-based integrated approach to testing and assessment (IATA) framework (Sachana et al. 2021). EFSA, in this context, conducted two IATA case studies to assess the applicability of the IVB DNT approach in the context of the European pesticide regulations (EU) 283/2013 and 1107/2009 (EFSA et al. 2021). A draft OECD GD for the evaluation of results obtained by the IVB DNT was recently published (OECD 2022a). Also, an IATA for non-genotoxic carcinogenicity is currently under development by an OECD expert group, including a comprehensive search for relevant assays suitable to address multiple biological elements to cover the large variety of non-genotoxic cancer mechanisms (Jacobs et al. 2020).

Sufficient identification of the observed biological perturbation and available NAMs that serve the purpose of addressing these mechanistic features are a pre-requisite for the development of a testing strategy. Investigating and expanding AOP networks to further explore the complexity of systems toxicology and to widen the range of toxicological space that can be covered by NAMs are major tasks in advancing mechanistic-based hazard assessment and paving the way for the development of appropriate test methods. In addition, such mechanistic information is particularly essential for MRA. As a general rule, mixture effects are most probable if the substances an individual is exposed to share the same molecular target and thus, behave as dilutions of one another. For regulatory MRA, substances which activate the same AOP are consequently evaluated together in a common assessment group. Moreover, with the continuous advancement in uncovering the interlinkage of AOPs in networks, substances with different MIEs that converge at a downstream KE to elicit a common adverse outcome can be included in the assessment (EFSA 2021). NAM data are, therefore, required to group similar substances based on their toxicodynamic and/or toxicokinetic properties in CAGs to allow for a robust MRA, predominantly conducted using single-substance *in vivo* data. Because the latter is often not available for each mixture component, NAMs (*in vitro* bioassays, IVIVE, PBK modeling) have recently been utilized not only for grouping but also in novel MRA strategies (Eccles et al. 2023; Ma et al. 2023). Apart from these component-based approaches to MRA, NAM-based testing and modelling regimes for mixture toxicity have been suggested to identify priority mixtures for further regulatory scrutiny (Braun and Escher 2023; Escher et al. 2022; Pistollato et al. 2021; Tralau et al. 2021). For example, within PANORAMIX an *in vitro* testing strategy combined with effect-directed analysis and chemical profiling will be applied to complex “real-life” mixtures to identify mixtures of concern (Escher et al. 2022). Other NAM-based strategies may be used to specifically investigate the interaction between mixture components to evaluate their potential for causing mixture toxicity. Accordingly, through NAMs (e.g. cellular assay profiles, omics) a toxicological profile or fingerprint of a regulatory well-characterized substance can be determined. Applying the same testing regime to combinations of the same substance with others in various mixtures may be an excellent way to gather valuable information on the interaction of the mixture components and their potential for eliciting mixture toxicity. Given the aforementioned constraints associated with *in vivo* testing, the potential benefits of NAMs for whole-mixture are evident.

The applicability of various omics technologies to uncover complex biological responses upon chemical perturbation has already been demonstrated, for instance using a metabolomics or transcriptomics approach in the context of read-across (Tate et al. 2021; van Ravenzwaay et al. 2017; Van Ravenzwaay et al. 2016). To facilitate the prediction of adverse outcomes based on omics-derived biomarker profiling, a human transcriptomics biomarker panel (S1500+) was generated (Mav et al. 2018). Predictive ToxicoGenomics Space (PTGS) modelling is yet another omics-driven concept for targeted and AOP-coupled mode-of-action analysis (Kohonen et al. 2017). Transcriptomics data and cytotoxicity data assessed by machine learning generated a 14-component, 1331 gene set-based, algorithm with superior prediction accuracy of drug-induced liver injury relative to other test methods (Kohonen et al. 2017). While perturbation of the transcriptome is considered a rather early event, changes in the metabolic profile are further downstream and consequently more strongly linked to a potential adverse outcome. With that in mind, the exercise was recently repeated to establish an equivalent panel for metabolic biomarkers (MTox700+) (Sostare et al. 2022). Under the Horizon2020 project PrecisionTox, the applicability of omics and molecular biomarkers will be further determined (Malinowska et al. 2022). The OECD EAGMST and Working Party on Hazard Assessment (WPHA) have joined forces to accelerate the regulatory use of omics (https://www.oecd.org/chemicalsafety/testing/omics.htm). Existing and newly developed NAMs should be collected and catalogued, clearly stating the information one can obtain from them including their individual strengths and limitations. Initiatives such as the Database on Alternative Methods (DB-ALM) produced by the EU Reference Laboratory on Alternatives to Animal Testing (EURL ECVAM) are a good starting point (European Commission 2019). Similarly, the US Toxic Substances Control Act (TSCA) mandates EPA to advance, validate and catalogue alternative methods.

### 3.2. Towards standardization and harmonization

While assessing animal data generated according to internationally accepted guidelines both in terms of interpreting the results and assessing the quality of the data is relatively straightforward, this process may be more difficult when looking at NAM-based data. With the exception of a few available guidelines, the majority of data generated using NAMs are neither conducted nor reported in a standardized manner. The amount of data generated from such non-guideline methods may nevertheless be precious for regulatory and scientific purposes at several levels of the safety assessment process. The reporting of non-guideline methods is, hence, a crucial aspect. To gain trust in NAMs in general and to facilitate the regulatory use of non-guideline methods in particular, multiple initiatives have published GDs and reporting templates. To promote reproducibility and robustness of cell culture assays, the first guidance on Good Cell and tissue Culture Practice (GCCP), published in 2005, already defined key performance principles for conducting such tests (Coecke et al. 2005). A revised version (GCCP 2.0) incorporating state-of-the-art *in vitro* technologies such as 3D culture systems, MPS, genetically modified cells and pluripotent stem cells, was recently released (Pamies et al. 2022). GDs on good *in vitro* practice, QSAR, and PBK models have been published by the OECD (OECD, 2007; OECD, 2018; OECD, 2021c). To tackle the issue of standardized reporting of information useful in a regulatory context, the OECD developed the GD 211 comprising recommendations as to how developers ideally should describe non-guideline *in vitro* test methods to make sure regulators can assess the quality of the data for regulatory purposes (OECD 2017). A test method description questionnaire (ToxTemp) was later developed to further specify these recommendations provided within the GD 211 and to improve understanding as to why regulators need such information (Krebs et al. 2019). Recently, the OECD, driven by the Joint Research Centre (JRC), has developed a harmonized and internationally agreed template for reporting mechanistic information of toxicity across and beyond apical endpoints, called OHT 201 (https://www.oecd.org/ehs/templates/harmonised-templates-intermediate-effects.htm). As envisaged also in the EU Chemicals Strategy for Sustainability (CSS), this template would allow relevant academic data to be transferred in a standardized format in order to be used for safety assessment. Meanwhile, EAGMST is also advancing a formal OECD Omics Reporting Framework (OORF) that is applicable for many types of omics data. Currently, the OORF includes modules relevant to transcriptomics and metabolomics toxicology studies with flexibility to incorporate other omics data types (such as proteomics) in the future (Harrill et al. 2021). More general approaches toward Good In Vitro Reporting Standards are on the way (Hartung et al. 2019).

Making use of standardized hazard reporting would allow regulators to quickly grasp methodological details and results to interpret data and draw conclusions on the quality and usefulness of the information, and scientists to effectively replicate them. Moreover, it supports the dissemination of new methods via structured databases. Ideally, standardized reporting and data management should follow the FAIR principles, i.e. Findable, Accessible, Interoperable and Reusable, to enable machine-actionability (Wilkinson et al. 2016). The exploitation of a solid data infrastructure will be vital for achieving the much desirable data-centric shift. In this regard, a second forward-looking definition of FAIR is “Findable AI Ready” (Scheffler et al. 2022). Should the above steps be implemented promptly and correctly, the advantages would be two-fold: current data sets based on animal studies would benefit from being FAIR and AI ready, and the regulatory risk assessment of the future would be able to make use of AI for data analytics, interpretation, management, integration and sharing. The potential of AI in the analysis of a large amount of data generated by these methods is increasingly recognized (Hartung and Tsatsakis 2021; Luechtefeld et al. 2018).

In addition, comprehensive regulatory guidance on the use (i.e. processing, interpretation, storage and curation) of NAM data for decision-making purposes is urgently needed before alternative approaches can be routinely and confidently used in risk assessment. For instance, while standards were agreed or developed for the collection and curation of omics data, data processing and interpretation still suffer from the lack of standardization of protocols (EFSA 2018; 2022). A recently published drafting framework on how to evaluate NAMs for human safety assessment attempts to provide a systemic approach to determine the applicability of a given NAM for various regulatory purposes (Parish et al. 2020). Others have come forward with a proposal for a framework specifically tailored to the implementation of NAMs under REACH (Ball et al. 2022). In an early stage of transitioning from the traditional animal-based approach to a system that is mostly grounded in NAMs, all information needs to be considered in a weight-of-evidence (WoE) approach (Linkov et al. 2015). Integrating various sources of information (human, animal, non-animal) in a coherent way requires comprehensive guidance and possibly a framework as well (Caloni et al. 2022).

### 3.3. Validation

For a wider acceptance of NAMs in regulatory toxicology, it is imperative to demonstrate that a given alternative method is not only robust and reproducible but also that it is biologically relevant and fit for its intended purpose (ICCVAM 2018). For instance, consensus is needed that certain information derived from NAM data is indicative of an adverse outcome likely to occur in humans. According to the OECD, validation of NAMs is needed to be covered by the Mutual Acceptance of Data (MAD) agreement. With the OECD GD 34, comprehensive guidance on the validation and acceptance of NAMs for regulatory purposes was made available as early as 2005 (OECD 2005). Several NAMs have already been validated and transformed into an OECD TG (Fig. 2). However, for some of these methods it took up to 20 years from invention to regulatory acceptance. Given the speedy development of an ever-growing number of methods, it becomes clear that the process of validation needs to undergo a paradigm change to allow for a more timely acceptance of NAMs that can be accepted by regulatory authorities (Burgdorf et al. 2019). Several options are considered in this context, *inter alia* the introduction of performance-based TGs, the use of hallmark or reference substances, case studies or a combination of these approaches.

Performance-based TGs like OECD TG 455 or TG 493 require performance standards that allow regulators to check if a new test method performs similarly to an existing one. Such performance standards are available for a number of endpoints (OECD, 2015; OECD, 2019; OECD, 2022b). Generally, they allow for implementation of methods that are similar to existing *in vitro* methods (e.g. additional estrogen receptor transactivation or binding assays) more quickly, while they do not replace *in vivo* assays per se.

As the aim of toxicity testing is generally to identify hazards and classify accordingly, hallmark or reference substances would help developing alternative methods by using them standardly as positive controls. Such substances have been extensively studied and classified for a specific hazard class (e.g. sensitization or irritation) or otherwise ascertained toxicity profile. Once agreed, hallmark substances, same as performance standards, would accelerate the acceptance of alternative methods, while also allowing for the introduction of new tests.

As an intermediate step, case studies can foster standardization potentially leading to the development of TGs for NAMs. Especially if conducted in parallel with traditional and alternative methods during a transitional period, they could help demonstrate the applicability domain of NAMs and the feasibility of a NAM-based assessment as well as promote concerted engagement of regulatory and research actors. The OECD has launched the IATA Case Studies Project (https://www.oecd.org/chemicalsafety/risk-assessment/iata-integrated-approaches-to-testing-and-assessment.htm) to promote the conduct of case studies exploring the regulatory use of NAM-based IATAs. Several case studies have also been carried out under the EU-ToxRisk research project (Moné et al. 2020; van der Stel et al. 2021). Work is underway by EPAA to start a User Forum on NAMs where industry and regulators jointly discuss NAM-based case studies. While in general a single case study is not capable of replacing validation, a combination of several case studies that all make use of similar methods to investigate the same type of hazard can increase the scientific and regulatory confidence.

**Fig. 4.**
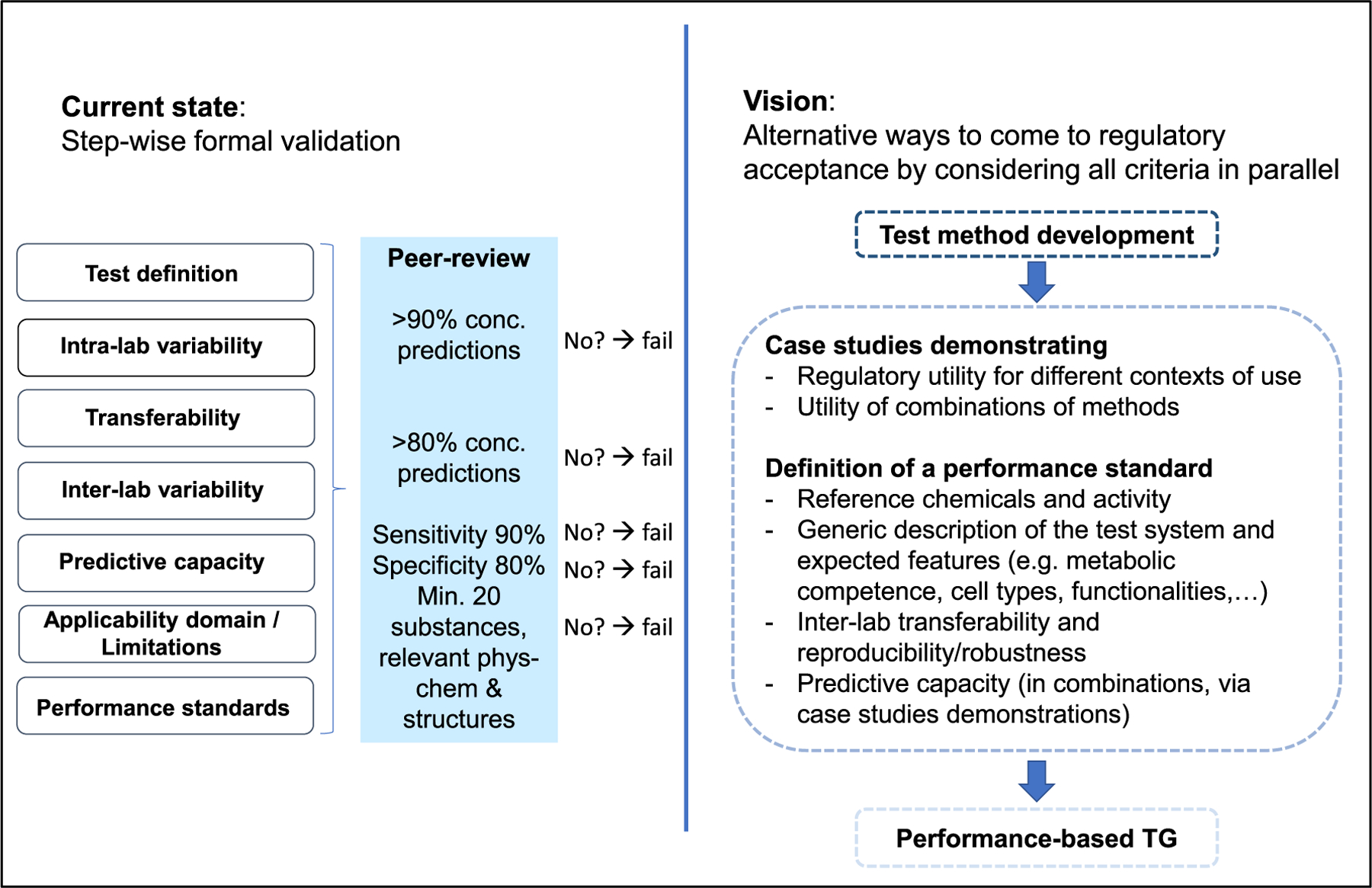
Current vs. envisioned approaches to NAM validation for a timely, yet robust, acceptance of NAMs by regulatory authorities.

The formal validation concept has been strongly influenced by the European Centre for the Validation of Alternative Methods (ECVAM; now transformed into the EU Reference Laboratory for alternatives to animal testing EURL-ECVAM), which evaluated NAMs mainly for use in the testing of industrial chemicals and pesticides. Other validation concepts and cultures have been developed in Europe in other fields (e.g. drug safety or food safety), and a large bandwidth of approaches is used in other geographic regions of the world. In the past 20 years, it has become clear that many strategies can be chosen for evaluating the performance, predictivity and relevance of a method (Patterson et al. 2021). Two particularly important new concepts are mechanistic validations (Hartung et al. 2013b), and the adaptation of validation requirements to the use of a NAM. The latter approach assumes that a NAM shows high readiness for e.g., screening and prioritization of a group of chemicals, but less readiness for setting regulatory reference values (Bal-Price et al. 2018; Pallocca et al. 2022). An alternative approach to validation is illustrated in Fig. 4.

### 3.4. Knowledge transfer and building confidence

In all cases, training and knowledge transfer are essential to overcome differences in expertise amongst different types of stakeholders. For example, constant communication between method developers and risk assessors aimed at demonstrating validity of NAMs can help foster a shift in mindset from apical effect demonstration in animals towards mechanistically informed, NAM-based hazard prediction in humans. Finally, education at earlier or other stages in the academic curriculum could also be a feasible way for building trust amongst stakeholders. In this regard, the EURL ECVAM has been engaged in education and training activities directed towards all stakeholders active in the field of regulatory toxicology (from wet lab scientists to EU Member State competent authorities: https://ec.europa.eu/jrc/en/eurl/ecvam/knowledge-sharing-3rs/education-and-training), as well as introducing education of the 3Rs to teachers in primary and secondary school, and at universities. The US EPA offers comprehensive training resources on various topics related to NAMs (https://www.epa.gov/chemical-research/new-approach-methods-nams-training). EU-funded projects such as SEURAT-1 and EU-ToxRisk have trained hundreds of young scientists in NAMs as well, and the Horizon 2020 ASPIS cluster and the Horizon Europe PARC initiative are continuing this effort. The need for such educational offers is illustrated by more than 15,000 active learners for new approach classes in just over three years on COURSERA.

### 3.5. Changes in regulation

There is a need for improvement in OECD TG development and approval to overcome the lack of standardized methods yielding data that can be made subject to MAD. In addition, regulation (e.g. based on classification and labelling) has to change at a global level, for example by updating the GHS with criteria to classify substances as carcinogenic or toxic to reproduction based on NAMs, once corresponding NAMs are available. One of the major obstacles for making the best use of NAMs according to the state of science is that current regulations especially in the EU usually explicitly ask for *in vivo* tests, with limited flexibility to do otherwise. This does not only apply to situations where NAM-based data would allow for equally protective assessment, but also hampers their application where they could potentially outperform the existing assays. Regulatory toxicology currently deals with this by retrospective guideline adaptation. However, this process has proven to be slow and is too inflexible to make the most out of newly available technologies. Instead of defining an assay, it would hence be far more efficient to lay out the regulatory information needs for addressing a given regulatory concern. In doing so, flexibility would improve without forfeiting the existing high level of protection. The aim should be no less than a systematic and concerted analysis of the guidelines available with the aim of rephrasing the respective protection goals in a result-oriented instead of an assay-based manner. The situation is comparable to the problem of assay validation where the overwhelming number of new assays has led to the concept of performance-based validation abandoning the idea of fully validating each assay anew (Fig. 4). This could easily be achieved by means of high-expertise working groups composed of regulators, agencies and leading academic colleagues in the field working together. In conjunction with the ongoing activities under ASPIS, EPAA, Horizon Europe, PARC, and other programs worldwide, this would allow for the biggest fundamental shift in chemical risk assessment seen for decades. In the US, the Interagency Coordinating Committee on the Validation of Alternative Methods (ICCVAM) has led several efforts to catalogue and define regulatory needs, and associated opportunities for NAMs, for various toxicity endpoints both within the US and globally (Choksi et al. 2019; Daniel et al. 2018; Strickland et al. 2018).

In Europe, changes in the legislations are initiated by the European Commission and the Member States. Hence, while regulators and the scientific community can prepare the scientific ground for an increased uptake or use of NAMs in a regulatory context, animal testing will remain mandatory as long as data requirements within legislation are not amended. On the other hand, while it will be a political decision to phase out animal testing it seems wise to be prepared. For this to happen, development and validation of NAMs, as well as assessment strategies built on NAMs, must accelerate to keep pace. But this alone will not suffice: also a clear strategy for the process of transitioning to a future framework of Next-Generation Risk assessment (NGRA) needs to be developed. Notably, beside the scientific aspects of such a strategy, outlined in the following section, also a strategic roadmap laying out the detailed process of making the legislation fit for accepting such a framework need to be elaborated in parallel.

## 4. Next-generation risk assessment (NGRA)

As mentioned in the introduction, leading experts in the NAM field were asked during the closed workshop to briefly elaborate on their personal vision for the future of chemical risk assessment during the closed workshop. While a gradual but steady uptake of NAMs into the chemical risk assessment was endorsed by all participants, a broader spectrum of opinions was voiced on the conceptual question of how exactly the chemical risk assessment of the future should accommodate or revolve around NAMs.

Recurrent key aspects are summarized here. Above all, the introduction of an integrated tiered approach, starting with a NAM-based biological activity screening across a broad toxicological space, yielding alerts or categories for follow-up testing or regulatory decision-making was mentioned in particular. Improving the understanding of human relevance and evaluating the performance of both traditional methods and NAMs accordingly were highlighted. As a result, the best performing method(s) for a given purpose/endpoint should always be considered and preferred in a flexible framework that makes the uptake of innovation easier. Given that NAM data provide different information, apical animal endpoints must be redefined and, in some cases, might even need to be abandoned in favor of a system that is driven by the analysis of the perturbation of biological mechanisms relevant to humans. As opposed to the traditional one-by-one approach, assessing combined exposures with the help of NAMs should be routinely included, where applicable to account for realistic exposure scenarios. While still challenging in some aspects, there was agreement that the ultimate goal should be a system that is protective, predictive and probabilistic (Maertens et al. 2022).

To collectively and proactively engage in shaping the future risk assessment, the establishment of a regular forum of public, private and political stakeholders (e.g. academic institutions, funding agencies, regulatory agencies, risk assessors, industry) was suggested for developing a concrete roadmap. This task has been taken up by the just-started European PARC project which is unique in that it includes the scientific as well as the regulatory community but also the leading regulatory agencies, ministries and stakeholders from the European Commission.

The first reference to Next-Generation Risk Assessment (NGRA) dates back to 2010, when the US EPA initiated the multi-year “NexGen” program with the aim of evaluating new molecular, computational, and systems biology-informed approaches and developing a new paradigm for the next generation of risk science (Cote et al. 2012). NGRA has generally been described as an exposure-led and hypothesis-driven approach that integrates *in silico*, *in chemico* and *in vitro* methodologies with use case information to foster a risk assessment that is more human-relevant. The International Cooperation on Cosmetics Regulation (ICCR) first explored the possibility to apply such concepts to real-life assessments as well as to agree on and outline the principles for incorporating NAMs into an integrated strategy for risk assessment of cosmetics ingredients (Dent et al. 2018). In a tiered and iterative framework, several non-animal methods are combined and individual uncertainty sources characterized in a structured and weighted manner (Bernauer et al.; Moné et al., 2020; van der Ven et al., 2020; Pallocca et al., 2022), with the overarching aim to prevent harm.

## 5. Conclusion

There is broad consensus among the scientific as well as a relevant part of the regulatory community that the use of NAMs and NGRA in regulatory risk assessment offers a great opportunity to further advance the protection of human health, be it by improving regulatory decision-making within set endpoints or approaches or by providing the tools for addressing new toxicological challenges. The implementation of NAMs clearly has the potential to accelerate the generation and interpretation of data. This would be beneficial to tackling the aforementioned performance issues of the current system and ultimately reducing the overall uncertainty with regard to chemical safety as a whole, associated with, for instance, a large number of poorly tested or even untested REACH chemicals on the market (Schaafsma et al. 2009). Yet, addressing the conceptual issues elaborated here will be a prerequisite for making use of NAMs or NAM-based approaches within the current system. In particular, it will require us to consider a fundamental mindset change regarding how we can handle endpoints and assays in a more flexible, NAM-ready way without compromising the level of protection offered by the current paradigm. The justification of the current, rather rigid legal requirements is historical and lies within the special socio-political remit of regulatory toxicology, i.e. health protection of the general public. First, there are obvious issues such as conservatism, scientific rigor and reliability that automatically arise when public health is involved. However, there also is a non-scientific requirement that results and decisions must follow predictable and legally sound procedures. Consequently, data requirements and assays as well as the approaches and assessments they feed, need to be highly standardized scientifically, but also in terms of administration. This makes the system inherently more resistant to change and methodological adaptation, for better or worse. Nearly all current frameworks and guidelines by design foresee taking up new assays as well as adaptation to the state of science.

These requirements and formal procedures were designed with a system in mind where assay readouts were expected to cover the systemic features of a particular endpoint, be it as a whole or in large parts. The other inherent assumption made is that every new method should have to live up to the existing animal data as gold standard. In contrast, the average NAM usually addresses certain key aspects of an endpoint and often does so using human biology-based molecular data, not systemic physiological readouts. Both aspects, i.e., the fragmentation of information and the human focus of the systems and models used result in limited compatibility with existing regulatory systems. To resolve this problem is far from trivial, as these hurdles apply long before any meaningful discussion on assay performance or interpretation will take place. This, therefore, is currently one of the main obstacles in making regulatory use of the best state of science.

To make the system fit for the challenges of the 21st century, we therefore suggest the following three steps for moving forward.

1. Risk assessment should be re-designed towards “NAM-readiness”, as the expectation that NAMs fit into the established framework by addressing existing regulatory endpoints in an animal-free manner is misleading. NAM data simply provide information of a different kind, which can be as informative and potentially more relevant for human health risk assessment when compared to their traditional *in vivo* counterparts. At the same time any proposed change, of course, will need to feature compatibility with basic legal requirements and the interlinkages of the framework in place. The apical endpoints in traditional testing should be critically evaluated under the remit of a performance-based system, which is driven by the assessment of biological human mechanism perturbation measured by means of fit-for-purpose methods. By doing so, the legislation would shift from the current animal-based, and partly black box, paradigm to a mechanistically informed, truly human-relevant probabilistic NGRA eventually. This requires critically assessing and comparing performance standards of new and existing methods as well as systematical appraisal and rephrasing existing testing requirements in a method-open manner.
2. We need to rethink how we set out regulatory requirements. Given that toxicology is all about adversity, principally thinking in endpoints makes sense. However, it is time to enlarge this concept by seeing and treating endpoints as physiological manifestations of the underlying pathways and cellular and molecular events. In order to avoid the aforementioned problems, the concept of prescribing fixed assays should gradually be developed further into providing more flexible toolboxes of different test methods/testing approaches, which nevertheless require standardization and harmonization. Future efforts should also focus on optimizing current AOPs, both individually and in networks, not only for quantification purposes, but also to include homeostatic feedback/forward loops and eventually distinguish adaptation from adversity.
3. We have to abstain from the default of making the existing assays a gold standard, but keep an open and performance-oriented perspective instead. This is also necessary to avoid amplification of assay fallacy. Every assay or system has limitations – benchmarking alternative systems to the performance of existing systems will by design risk also amplifying their very limitations. This report has outlined strategies and solutions for dealing with this, which, however, will take concerted efforts by all stakeholders involved.

Not acting is not an option. We would therefore suggest using the upcoming regulatory research initiatives such as PARC in Europe for taking up these issues in order to promote real change. This also includes the need for developing a strategic framework for making the legal frameworks “NAM-ready” step by step, an aspect which has been underrepresented in previous research initiatives. In addition, we would like to suggest the establishment of working groups for the evaluation and re-description of regulatory testing requirements. Ideally, these should consist of relevant and methodologically up-to-date experts from both the scientific and regulatory community, including national and international agencies, to foster an open-minded and productive dialogue. This could include collaboration between industry and regulators to address the risk of rejection and legal liabilities, ultimately incentivizing the uptake of NAMs. To allow this change to happen, Fig. 3 illustrates key individual and collaborative responsibilities of all stakeholders involved in the transition, with a tentative timeline depicted in Fig. 2.

## Data Availability

No data was used for the research described in the article.

## Abbreviations:

AI: Artificial Intelligence
AOPs: Adverse Outcome Pathways
APCRA: “Accelerating the Pace of Chemical Risk Assessment”
BfR: German Federal Institute for Risk Assessment
BMD: Benchmark Dose Modelling
CSS: Chemicals Strategy for Sustainability
DA: Defined Approaches
DART: Developmental And Reproductive Toxicology
DB-ALM: Database On Alternative Methods
DIP: Data Interpretation Procedure
DNT: Developmental Neurotoxicity
EAGMST: Extended Advisory Group for Molecular Screening And Toxicogenomics
ECHA: European Chemicals Agency
ED: Endocrine Disruption
EDSP: Endocrine Disruptor Screening Program
EFSA: European Food Safety Authority
EPAA: European Partnership for Alternative Approaches to Animal Testing
EURL ECVAM: EU Reference Laboratory on Alternatives to Animal Testing
FAIR: Findable, Accessible, Interoperable and Reusable
GCCP: Guidance on Good Cell And Tissue Culture Practice
GD: Guidance Document
GD-GIVMP: Guidance Document on Good In Vitro Method Practices
HBGV: Health-Based Guidance Value
HTS: High-Throughput Screening
IATA: Integrated Approaches to Testing And Assessment
ICCR: International Cooperation on Cosmetics Regulation
ICCVAM: Interagency Coordinating Committee on the Validation of Alternative Methods
IRS: Integrated Regulatory Strategy
IVB DNT: In Vitro Battery for DNT
JRC: Joint Research Centre
KEs: Key Events
LOAEL: Lowest Observed Adverse Effect Level
MAD: Mutual Acceptance of Data
MIEs: Molecular Initiating Events
MOA: Mode Of Action
MPS: Microphysiological Systems
NGRA: Next-Generation Risk Assessment
Ngtx carc: Non-Genotoxic Carcinogens
NTA: Non-Targeted Analysis
OECD: Organisation for Economic Co-Operation and Development
OORF: OECD Omics Reporting Framework
PARC: “Partnership for the Assessment of Risks From Chemicals”
PBK: PhysiologyBased Kinetic Modelling
POD: Point Of Departure
PTGS: Predictive Toxicogenomics Space
qAOP: Quantitative Adverse Outcome Pathway
QIVIVE: Quantitative In Vitro *to In Vivo* Extrapolation
QSAR: Quantitative Structure-Activity Relationship
RMSE: Root-Mean-Square Error
SIRs: Standard Information Requirements
SSA: Suspect Screening Analysis
STOTs: Specific Target Organ Toxicities
TGs: Test Guidelines
TSCA: Toxic Substances Control Act
UN GHS: Globally Harmonized System of Classification and Labelling of Chemicals of the United Nations
US EPA: United States Environmental Protection Agency
WOE: Weight Of Evidence
WPHA: Working Party on Hazard Assessment
